# Preferences for childbirth delivery and pain relief methods among pregnant women in Vietnam

**DOI:** 10.3389/fmed.2024.1290232

**Published:** 2024-01-30

**Authors:** Tham Thi Nguyen, Long Hoang Nguyen, Ha Thu Thi Nguyen, Vu Anh Trong Dam, Thuc Minh Thi Vu, Carl A. Latkin, Melvyn W. B. Zhang, Roger C. M. Ho, Cyrus S. H. Ho

**Affiliations:** ^1^Institute for Global Health Innovations, Duy Tan University, Da Nang, Vietnam; ^2^Faculty of Nursing, Duy Tan University, Da Nang, Vietnam; ^3^Department of Global Public Health, Karolinska Institutet, Stockholm, Sweden; ^4^Hanoi Obstetrics and Gynecology Hospital, Hanoi, Vietnam; ^5^Institute of Health Economics and Technology (iHEAT), Hanoi, Vietnam; ^6^Bloomberg School of Public Health, Johns Hopkins University, Baltimore, MD, United States; ^7^Lee Kong Chian School of Medicine, Nanyang Technological University Singapore, Singapore, Singapore; ^8^Department of Psychological Medicine, Yong Loo Lin School of Medicine, National University of Singapore, Singapore, Singapore; ^9^Institute for Health Innovation and Technology (iHealthtech), National University of Singapore, Singapore, Singapore

**Keywords:** childbirth delivery, pain relief, pregnant women, women’s health, Vietnam

## Abstract

**Background:**

Understanding childbirth delivery and pain relief method preferences is important as a part of the shared decision-making process between pregnant women and health professionals. This study aimed to examine the preferences for childbirth delivery modes and pain relief methods and factors related to these preferences among pregnant women in Vietnam.

**Methods:**

A cross-sectional survey on pregnant women was conducted in two obstetrics hospitals in Vietnam. Face-to-face interviews using a structured questionnaire were performed to collect information about sociodemographic characteristics, pregnancy characteristics, preferences for different childbirth delivery modes, and pain relief methods. Multivariate logistic regression was employed for determining associated factors with the preferences.

**Results:**

Of 576 pregnant women, 34% of participants preferred cesarean section. Most of the sample did not have any preferences for specific pharmacological pain relief methods (70.1%), while support from partner/relatives was the most preferable non-pharmacological method (61.3%), following by water birth (11.1%) and acupuncture (9.9%). Desire to have another baby, relatives’ experience, selection date of birth, and instrumental social support were major drivers of the cesarean section selection. This preference was an important factor in the preference for pharmacological pain relief. Meanwhile, high levels of informational and emotional support were associated with non-pharmacological method preference.

**Conclusion:**

This study highlighted a high preference rate for cesarean section in urban pregnant women in Vietnam. Holistic approaches from family, health facility, and policy should be performed to diminish the cesarean rate preference and promote the use of non-pharmacological pain relief methods during birth.

## 1 Introduction

The choice of childbirth delivery method is one of the most crucial concerns of women before and during pregnancy (1, 2). Vaginal delivery has advantages including less postpartum pain, fewer labor and delivery complications, requiring a short hospital stay, and the immediate ability to breastfeed (3). However, this method cannot be performed if the pregnant women have some specific conditions such as a large baby, head-pelvis incompatibility, or fetal distress, where cesarean delivery is preferable as a lifesaving method for both mothers and children (4, 5). In general, cesarean deliveries are recommended that should be performed for medical reasons, not due to preferences (5). Because, the cesarean section may pose great complications like hysterectomy, abnormal placentation, uterine rupture, or stillbirth, as well as increase the risk of preterm birth in the next pregnancies (6).

The rate of cesarean section is rising by over 15% in many nations worldwide exceeding the cesarean rate recommended by World Health Organization (WHO) (7). Particularly, global literature recognized that overall, approximately 21.1% of pregnant women preferred cesarean section (8), and this rate varied across countries, especially high-income countries such as 34% in Australia, 32.2% in the United States, 26.2% in the United Kingdom, 26% in Canada, and 50.4% in Turkey (8). Furthermore, this review has indicated that several factors such as fear of labor pain and safety of baby, previous birth experience, fear of postnatal complications, consultation of health professionals, social and cultural influences, and access to information are recognized pervasive causes of this phenomenon (8). Besides, the preference for cesarean section is more common among women aged over 30, living in urban settings, having higher socioeconomic status, and fearing vaginal delivery than others (9, 10). This preference is also observed in pregnant women with high gestation weeks. Particularly, the rate of preference for cesarean section changed from 14% in the first trimester to 21% in the third trimester (11).

Another part of decision-making during childbirth delivery is the pain relief method. There are two types of pain relief methods during the labor process including pharmacological and non-pharmacological interventions (12). Pharmacological interventions consisted of the use of epidurals and opioids, while non-pharmacological interventions included massage and relaxation methods such as yoga, music, breathing techniques, shiatsu, reflexology, and others. Several previous publications proved that the main reason for women’s preference for pharmacological and cesarean section is their fear of suffering severe pain during childbirth and lack of knowledge regarding the possible adverse consequences of these methods after delivery (8, 13). Particularly, although pharmacological methods may more effectively reduce labor pain, they may have several negative side effects such as postnatal back pain and difficulty in breastfeeding (14). Meanwhile, non-pharmacological methods, which can be performed by the husband/relatives or healthcare workers not only can decrease labor pain but also reduce the risk of childbirth complications among those using analgesia (15). Moreover, these methods enhance bonding between pregnant women with midwives and obstetricians (14) as well as enhance the feeling of joyfulness and empower women in childbirth delivery (14), reduce childbirth fear (16), diminish the labor time and elevate the satisfaction after delivery compared to the pharmacological interventions (17, 18). Thus, timely providing appropriate pain relief methods is a critical factor in reducing the preference for cesarean delivery. Furthermore, women can find information related to pain relief methods in some sources such as family and friends, healthcare professionals, books, the internet, and antenatal classes before and during pregnancy to select preferable pain relief methods for themselves (19–21).

In Vietnam, the Ministry of Health enacted nationwide guidelines for reproductive health services in 2017 (22). However, these guidelines have mainly focused on medical techniques of childbirth and postnatal care for the mother as well as the child (22). There is no recommendation regarding the rate of the cesarean section, even though this method has been overused in Vietnam, particularly in urban areas (23). The rate of cesarean delivery has remarkedly increased from 9.9% in 2002 to 27.5% in 2021 (24). The cesarean section rate was higher in urban areas (38.5%) than that in rural areas (12.4%) (25). In Vietnam, only one qualitative study was performed to determine the perceptions of pregnant women on the factors associated with the significant increase of cesarean section (4) and the findings indicated that fear of labor pain was the main reason for this phenomenon. Besides, there is a lack of quantitative evidence about this issue. Moreover, the preference for pain relief methods among pregnant women has not yet been investigated in Vietnam. To date, all obstetrics hospitals in Vietnam provided pharmacological interventions to release the labor pain according to the guideline, but non-pharmacological intervention services have not been yet mentioned or delivered. This study aimed to examine the preferences for childbirth delivery modes and pain relief methods and factors related to these preferences among pregnant women in Vietnam. The findings of this study would partly contribute to evaluating the feasibility and acceptability of non-pharmacological methods in labor pain relief.

## 2 Methods

### 2.1 Study design and participants

A cross-sectional study was conducted in two hospitals in Vietnam, including Hanoi Obstetrics & Gynecology hospital and Ca Mau Obstetrics & Pediatrics provincial hospital from January to February 2021. The eligibility criteria for participating as follows: (1) Aged 18 and older; (2) Agree to participate in research; (3) Planning to deliver in the selected hospital; (4) Being able to answer the interview questionnaire; and (5) Visit the hospital for regular antenatal care during the study period. Pregnant women were conveniently approached and recruited for this study when they visited these hospitals.

In this study, sample size computations were performed to estimate population proportion with a specified relative precision. We used confidence level (%) α = 0.05, expected population proportion *p* = 39% (26), and relative precision ε = 0.1. To prevent not responses or dropout, we added 15% of the sample size, resulting in the necessary sample size was 690 participants. At the end of the data collection process, 669 participants agreed to participate in this study (response rate = 95%). After excluding those not completing the questionnaire about delivery method, using pain relief with/ without medicine, the data of 576 participants were included in the data analysis process (completion rate 86.1%).

### 2.2 Data collection and measurement

In this study, a structured questionnaire was used for face-to-face interviews. To construct this research instrument, a standard procedure was applied. In the first stage, we carried out a systematic review to explore the gaps in research problems as well as important facets of the topic of interest from the previous evidence. From that, an initial research instrument was developed and covered all aspects of the topic of interest. In all stages of the research questionnaire development process, we invited several experts in obstetrics and gynecology, health services providers, and policymakers to jointly discuss and deliberate from the translating, rephrasing, piloting, as well as shortening the questionnaire.

The final version of the research instrument consists of five major components: 1) Demographic characteristics; 2) Pregnancy characteristics; 3) Preference for delivery method and reasons; 4) Preference for pain relief during and after delivery (with pharmacological and non-pharmacological interventions) and reasons, and 5) Social support. Furthermore, before the official data collection processing, we piloted this questionnaire among 30 pregnant women of different ages, periods of pregnancy, and living locations to test the language, logical order, and meaning of each question of the research instrument once again. After the research instrument had been revised, we removed all of the piloted data. During the data collection process, face-to-face interviews with participants were conducted within 15–20 min by research team members who were well-trained to use the research instrument.

#### 2.2.1 Demographic and pregnancy characteristics

Participants were asked a number of questions about their information, including age, education, occupation, living arrangement, and monthly household income. Pregnancy characteristics consisted of the number of pregnancies, complications of pregnancy, source of maternal care information (health professionals, internet/social networks, friends/relatives, radio & television, smartphone applications, newspapers & books, phone messages, or posters/banners) and desire to have another baby. Fear of childbirth was asked with 11-point rating scales from 0 “No fear” to 10 “Extreme fear.” We also asked pregnant women that whether they decided the day and time for childbirth for fortune or not.

#### 2.2.2 Preferences for childbirth delivery methods

To ask participants about the type of preferred delivery method, we used the question “What delivery method do you prefer during this pregnancy?” with 2 answer options (Vaginal delivery/Caesarean). Besides, other questions related to the reasons for choosing the type of delivery (Experience from a previous pregnancy/ Consultation of healthcare staff/ Experience from acquaintances, friends/ Consider my physical health/ Consider the cost/ Others), “Has your family members ever had a cesarean section?” or “Did you ever heard about cesarean section?” with answer options (Yes/No).

#### 2.2.3 Preferences for pain relief methods

A question “Which pharmacological method do you prefer for pain relief during childbirth delivery?” was used with different response options (e.g., Do not use/ Spinal anesthesia/ Epidural anesthesia/ Intravenous/ Intramuscular Labor Pain Relief/ Depends on the doctor). In addition, we asked the reasons for the preference (including experience from a previous pregnancy; experience from acquaintances/friends; consultation of healthcare staff; consider physical health; consider the cost; and others).

Another question, “Do you want to use non-pharmacological pain relief method?” was asked to learn about the non-pharmacological method that participants wanted to use such as relatives support and care during delivery, using water for pain relief, hypnotherapy for pain relief, acupuncture, acupressure pain relief, or transcutaneous electrical stimulation for pain relief.

Preferable methods of pain relief after childbirth were also asked participants with options including Do not use/ Epidural and continuous anesthesia for 48 h after surgery/ Infusion of pain relievers intravenously 6 h or vial/ Injections of pain relievers or morphine/ Put pain relief in the anus/ Depends on the doctor/ Others. The reason for this preference was also asked.

#### 2.2.4 Social support

The Perinatal Infant Care Social Support (PICSS) instrument was used to evaluate social support (27). This instrument consisted of 22 items and divided into four subscales: Informational support (7-item); Instrumental support (7-item); Emotional support (4-item); Appraisal support (4-item). A 4-Likert scale was used to respond for each item, from 1 “Totally disagree” to 4 “Totally agree.” The total score of each subscale was summed, and a higher score indicated higher social support. The Cronbach’s alpha was good at 0.85.

### 2.3 Statistical analysis

Stata software version 16 was used to analyze the data. Descriptive statistical analysis was performed. Chi-squared and Wilcoxon rank-sum tests were utilized to compare sociodemographic characteristics, pregnancy characteristics, reasons for preferring childbirth delivery methods, preferable pain relief methods between those preferring vaginal delivery and cesarean section. A value of p (p) <0.05 was considered statistically significant. Multivariate Logistic Regression models were employed to determine factors associated with the preferable types of delivery method and pain relief methods during childbirth. A forward stepwise selection was used to construct the reduced model, which only included independent variables having a value of p of log-likelihood ratio test less than 0.2.

## 3 Results

Table 1 shows that among 576 pregnant women, 34% of participants preferred cesarean section (95%CI = 30.1–38.1%), while 66.0% of them preferred vaginal delivery (95%CI = 61.9–68.8%).

**Table 1:** Preferable childbirth delivery method among pregant women (*n* = 576).

**Preferable childbirth delivery method**	***n***	**%**	**95% CI**
Vaginal Delivery	380	66.0	61.9–68.8%
Cesarean section	196	34.0	30.1–38.1%

Among 576 pregnant women, the mean age was 28.9 years (Table 2). Table 2 also shows that the significant differences between those preferring vaginal delivery and cesarean section were observed regarding age, living with parents-in-law, the number of pregnancies, having experience with threatened miscarriage during pregnancy, desire to have another baby, choosing a specific date and time for delivery, and fear of childbirth (*p* < 0.05).

**Table 2:** Demographic and pregnancy characteristics.

**Characteristics**	**Preferable childbirth delivery method**						
	**Vaginal Delivery**		**Cesarean section**		**Total**		***p* value**
	***n***	**%**	***n***	**%**	***n***	**%**	
**Total**	380	100.0	196	100.0	576	100.0	
**Province**							
Hanoi	227	59.7	116	59.2	343	59.5	0.90
Ca Mau	153	40.3	80	40.8	233	40.5	
**Education of mother**							
Secondary or below (graduated 9th grade or below)	48	12.7	26	13.3	74	12.9	0.89
High school (graduated 12th grade)	128	33.9	66	33.7	194	33.8	
College/University	57	15.1	34	17.3	91	15.9	
Postgraduate	145	38.4	70	35.7	215	37.5	
**Occupation**							
Farmer/Blue-collar worker	51	13.4	30	15.3	81	14.1	0.78
Public servant	36	9.5	14	7.1	50	8.7	
Office worker	115	30.3	61	31.1	176	30.6	
Housewife	117	30.8	64	32.7	181	31.4	
Others	61	16.1	27	13.8	88	15.3	
**Living arrangements**							
Parents	33	8.7	16	8.2	49	8.5	0.83
Parents in law	172	45.3	69	35.2	241	41.8	0.02
Partner, children	218	57.4	129	65.8	347	60.2	0.50
**Living location**							
Urban	222	58.4	103	52.6	325	56.4	0.18
Rural	158	41.6	93	47.4	251	43.6	
**Number of pregnancies**							
None	175	49.3	48	25.1	223	40.8	<0.01
One	143	40.3	99	51.8	242	44.3	
Two or more	37	10.4	44	23.0	81	14.8	
**Pregnancy complications**							
Threatened miscarriage	29	7.6	34	17.3	63	10.9	<0.01
Abnormal fetus	13	3.4	9	4.6	22	3.8	0.49
Local infections	6	1.6	2	1.0	8	1.4	0.59
Stress	6	1.6	3	1.5	9	1.6	0.97
Others	12	3.2	4	2.0	16	2.8	0.44
**Source of information**							
Internet/Social network	254	66.8	117	59.7	371	64.4	0.09
Health professional	237	62.4	124	63.3	361	62.7	0.83
Friends/Relatives	220	57.9	105	53.6	325	56.4	0.32
Smartphone application	107	28.2	50	25.5	157	27.3	0.50
Radio, television	103	27.1	48	24.5	151	26.2	0.50
Newspapers, book	81	21.3	42	21.4	123	21.4	0.98
Phone message	33	8.7	12	6.1	45	7.8	0.28
Poster/Banner	20	5.3	10	5.1	30	5.2	0.93
**Desire to have another baby**							
Yes	121	31.8	32	16.4	153	26.6	<0.01
No	83	21.8	66	33.8	149	25.9	
Unknown	176	46.3	97	49.7	273	47.5	
**Choosing specific date and time for childbirth delivery**							
Yes	11	2.9	52	26.5	63	10.9	<0.01
No	369	97.1	144	73.5	513	89.1	
	**Mean**	**SD**	**Mean**	**SD**	**Mean**	**SD**	**value of p**
**Age (years old)**	28.5	5.2	29.7	5.3	28.9	5.3	<0.01
**Monthly income (Unit: USD)**	596.6	472.1	619.3	647.1	604.4	538.2	0.68
**Fear of childbirth (0–10)**	5.8	2.4	5.1	2.6	5.6	2.5	<0.01
**Social support (PICSS)**							
Appraisal support (4–16)	12.3	1.4	12.3	1.3	12.3	1.4	0.89
Emotional support (4–16)	12.3	1.6	12.4	1.3	12.3	1.5	0.34
Informational support (7–28)	21.4	3.0	21.9	2.1	21.6	2.7	0.13
Instrumental support (7–28)	21.4	2.8	21.9	2.4	21.5	2.7	0.12

* PICSS: Perinatal Infant Care Social Support.

Table 3 indicates that out of the participants who preferred cesarean section, there were 66.3 and 99.5% of them had other family members undergoing the same delivery method and had heard/experienced the cesarean section method, respectively. In terms of the reason for delivery mode preferences, consultation of physicians (vaginal delivery: 57.6% and cesarean section: 51.0%) was the most common reason for both vaginal delivery preference and cesarean section preference, followed by considering health condition (vaginal delivery: 31.8% and cesarean section: 35.2%), and previous experience (vaginal delivery: 28.7% and cesarean section: 37.2%). The delivery methods among the participants were mostly decisions by themselves or their families. However, the proportion of participants preferring cesarean section and vaginal delivery based on the consult from others (such as physicians) was 10.2 and 2.4%, respectively.

**Table 3:** Reasons for the delivery mode preferences.

**Characteristics**	**Preferable childbirth delivery method**						
	**Vaginal Delivery**		**Cesarean section**		**Total**		***p* value**
	***n***	**%**	**n**	**%**	**n**	**%**	
**Family having person(s) undergoing cesarean section**	141	37.1	130	66.3	271	47.0	<0.01
**Heard about/Experience cesarean section**	356	93.7	195	99.5	551	95.7	<0.01
**Reasons for preference**							
Consultation of physicians	219	57.6	100	51.0	319	55.4	0.13
Consider health condition	121	31.8	69	35.2	190	33.0	0.42
Previous experience	109	28.7	73	37.2	182	31.6	0.04
Friends/relatives’ experience	75	19.7	27	13.8	102	17.7	0.08
Consider cost	41	10.8	2	1.0	43	7.5	<0.01
Others	2	0.5	11	5.6	13	2.3	<0.01
**Decision-makers**							
Self	368	96.8	174	88.8	542	94.1	<0.01
Family	3	0.8	2	1.0	5	0.9	
Others	9	2.4	20	10.2	29	5.0	

Table 4 shows that regarding pharmacological methods, most of the sample did not have specific methods (70.1%) and based on consultation of physicians (69.8%), while 11.6% of pregnant women reported that they did not need these methods. Meanwhile, regarding non-pharmacological methods, support from partner/relatives was the most preferable method (61.3%), following by water birth (11.1%) and acupuncture (9.9%).

**Table 4:** Preferences for pain relief methods.

**Characteristics**	**Preferable childbirth delivery method**						
	**Vaginal Delivery**		**Cesarean section**		**Total**		***p* value**
	***n***	**%**	**n**	**%**	**n**	**%**	
**Preferable pharmacological method**							
No need	59	15.5	8	4.1	67	11.6	<0.01
Depends on the doctor	267	70.3	137	69.9	404	70.1	0.93
Epidural anesthesia	41	10.8	19	9.7	60	10.4	0.68
Spinal anesthesia	7	1.8	34	17.3	41	7.1	<0.01
Intravenous morphine-like pain relief	12	3.2	8	4.1	20	3.5	0.57
**Reasons for preference**							
Consultation of physicians	258	67.9	144	73.5	402	69.8	0.18
Consider health condition	60	15.8	58	29.6	118	20.5	<0.01
Previous experience	57	15.0	21	10.7	78	13.5	0.15
Friends/relatives’ experience	43	11.3	12	6.1	55	9.5	0.04
Consider cost	43	11.3	5	2.6	48	8.3	<0.01
Others	4	1.1	2	1.0	6	1.0	0.97
**Preferable non-pharmacological method**							
No need	99	26.1	59	30.1	158	27.4	0.30
Support from partner/relatives	239	62.9	114	58.2	353	61.3	0.27
Water birth	47	12.4	17	8.7	64	11.1	0.18
Acupuncture	44	11.6	13	6.6	57	9.9	0.06
Hypnotherapy	28	7.4	10	5.1	38	6.6	0.30
Transcutaneous electrical nerve stimulation	17	4.5	8	4.1	25	4.3	0.83
**Preferable pharmacological method after childbirth delivery**							
No need	41	10.8	9	4.6	50	8.7	0.01
Depends on the doctor	289	76.1	135	68.9	424	73.6	0.06
Epidurals for 48 h after surgery	28	7.4	36	18.4	64	11.1	<0.01
Use rectal analgesia	38	10.0	20	10.2	58	10.1	0.94
Infusing pain relievers intravenously	8	2.1	8	4.1	16	2.8	0.17
Injecting morphine-like pain relievers	5	1.3	1	0.5	6	1.0	0.37
Others	3	0.8	1	0.5	4	0.7	0.70

Results of multivariate logistic regression are shown in Table 5. Only variables selected via the stepwise process were presented. Women desiring to have another baby (OR = 0.40, 95%CI = 0.20–0.82), receiving information from the Internet/Social network (OR = 0.55, 95%CI = 0.33–0.92) and having a higher level of childbirth fear (OR = 0.83, 95%CI = 0.75–0.92) were less likely to prefer cesarean section. Meanwhile, women having family members undergoing cesarean section (OR = 6.37, 95%CI = 3.79–10.71) or hearing about cesarean section (OR = 8.40, 95%CI = 1.09–64.56) were more likely to prefer cesarean delivery. When the score of instrumental support increased by 1 point, they tended to have higher odds of preferring cesarean sections about 1.11 times (OR = 1.11, 95%CI = 1.02–1.21). Women intending to choose a specific date and time for childbirth delivery had a strong likelihood of preferring cesarean section compared to those not intending (OR = 13.67, 95%CI = 5.36–34.92).

**Table 5:** Factors associated the delivery method preferences among pregnant women.

VARIABLES	Childbirth delivery method preference (Vaginal delivery = 0 / Cesarean section = 1)	
	OR	95%CI
**INDIVIDUAL CHARACTERISTICS**		
Province (Ca Mau vs. Hanoi – ref)	2.89**	1.43; 5.85
Desire to have another baby (vs No – ref)		
Unknown	0.73	0.36; 1.48
Yes	0.40***	0.20; 0.82
**PREFERENCE FOR DELIVERY**		
Family having person(s) undergoing cesarean section (Yes vs No – ref)	6.37***	3.79; 10.71
Heard about/Experience cesarean section (Yes vs No – ref)	8.40**	1.09; 64.56
Source of information from Internet/Social network (Yes vs No – ref)	0.55**	0.33; 0.92
Fear of childbirth (per score)	0.83***	0.75; 0.92
Choosing specific date and time for delivery (Yes vs No)	13.67***	5.36; 34.92
**THE PERINATAL INFANT CARE SOCIAL SUPPORT**		
Instrumental support (per score)	1.11**	1.02; 1.21
Appraisal support (per score)	0.83	0.65; 1.07

*** *p* < 0.01, ** *p* < 0.05, * *p* < 0.1.

Table 6 illustrates that pregnant women desiring to have another baby tended to have higher odds of preferring non-pharmacological relief methods compared to those who have no desire (OR = 2.31; 95%CI = 1.22–4.37). In terms of source of health information, people who received information from friends or relatives were more likely to have higher odds of preferring both pharmacological relief methods (OR = 2.19, 95%CI = 1.02–3.68) and non-pharmacological relief methods (OR = 2.21; 95%CI = 1.27–3.84). Furthermore, people using smartphone applications as a health information resource tended to prefer non-pharmacological relief methods (OR = 2.94; 95%CI = 1.03–3.64). Regarding social support, when the score of informational support (OR = 1.28; 95%CI = 1.09–1.51) and emotional support (OR = 1.32; 95%CI = 1.09–1.60) increased by 1 point, they were also more likely to prefer non-pharmacological relief methods.

**Table 6:** Factors associated with preferences for pain relief methods with or without medicine.

VARIABLES	Preferring any pharmacological methods during delivery (No = 0/Yes = 1)		Preferring any non-pharmacological methods during delivery (No = 0/Yes = 1)	
	OR	95%CI	OR	95%CI
**Individual characteristics**				
**Province (Ca Mau *vs* Hanoi – ref)**	19.71***	5.62; 69.12	8.22***	3.61; 18.73
**Occupation (*vs* Farmer/Blue-collar worker – ref)**				
Public servant			0.76	0.24; 2.43
Office worker			1.04	0.38; 2.82
Housewife			0.38**	0.14; 1.00
Others			0.44	0.16; 1.23
**Living arrangement (Yes *vs* No – ref)**				
Parents			0.53	0.23; 1.19
**Desire to have another baby (*vs* No-ref)**				
Unknown			2.84***	1.35; 5.96
Yes			2.31**	1.22; 4.37
**DELIVERY SERVICES**				
**Childbirth delivery method preference (Cesarean section *vs* Vaginal delivery - ref)**	4.21***	1.69; 10.50		
**Source of information (Yes *vs* No – ref)**				
Poster/Banner	0.19**	0.04; 0.85		
Friends/Relatives	2.19**	1.02; 4.68	2.21***	1.27; 3.84
Smartphone applications			1.94**	1.03; 3.64
Newspapers, books			1.83	0.87; 3.81
Radio, television	0.35**	0.14; 0.90		
Physicians	1.77	0.78; 4.02		
**The perinatal infant care social support**				
Informational support (per score)			1.28***	1.09; 1.51
Instrumental support (per score)			0.81**	0.68; 0.97
Emotional support (per score)			1.32***	1.09; 1.60

*** *p* < 0.01, ** *p* < 0.05, * *p* < 0.1.

## 4 Discussion

Study results suggested that although the majority of our pregnant women preferred vaginal delivery, the proportion of individuals having demands on cesarean section was high. In this study, more than a third of pregnant women preferred cesarean delivery, which was significantly higher than the overall preference rate with 15.6% (28), as well as this rate in other countries (5, 29–32). The differences might be due to different demographic characteristics and pregnancy status in these studies. In addition, our study was conducted in urban obstetrics hospitals; thus, the rate of preference for cesarean sections might be higher than in other studies. Global research also showed that the proportion of urban women who preferred this method was higher than that in rural areas (5, 28). However, this result seemed to be consistent with the proportion of cesarean section performance in urban Vietnam (38.5%) which was reported in the previous study (25).

The most common reasons for this preference included the doctor’s recommendation, considering own health and based on previous experience, which echoed previous research in Vietnam and other countries when revealing that these factors were also the main reasons for the woman’s request for cesarean delivery (4, 33–35).

Notably, our study indicated that fear of childbirth and desire to have another baby were negatively associated with the preference for cesarean section. This was contradicted with previous findings in other studies, which showed that fear of childbirth was a significant predictor for the cesarean delivery preference (36–38). However, several prior studies indicated that fear of childbirth did not have any association with this preference (10, 39). Reasons for this phenomenon are not clear, but we supposed that the fear of postpartum pain was an important factor in explaining this problem. Research in Japan showed that pregnant women were aware that postoperative pain after the cesarean section was much more severe than postpartum vaginal pain; hence, they preferred vaginal birth over cesarean section even if they could tolerate pain (39). In addition, we believed that this phenomenon could be justified based on the decision-making process of the pregnant woman and the physicians in the hospitals where we performed this study. In these hospitals, pregnant women were thoroughly consulted about the childbirth delivery process, including the advantages and disadvantages of each method during and after the delivery. For instance, the cesarean section may increase the risk of postpartum complications such as hysterectomy, abnormal placentation, uterine rupture, or stillbirth, as well as increase the risk of preterm birth in the next pregnancies (6). For women with a high degree of fear of giving birth and having a desire to have another baby, health professionals performed mindfulness therapies to help reduce anxiety, and stress for women as well as prevent health problems in the next pregnancy.

Results of our study were similar to previous studies showing that women whose relatives have had a cesarean section or have heard and experienced a cesarean section were more likely to prefer the cesarean delivery method when giving birth (5, 40–42). One important socio-cultural factor we found in the study was that the choice of a specific date and time for childbirth was strongly associated with the preference for a cesarean section. This result reinforces the findings of previous studies in Vietnam and China (4, 11). In fact, in Vietnam, in some pregnant women and their families, the choice of birth date plays an important role when they conceive that having a baby at a good date and time of birth can help their family get fortune, success, and happiness as the baby harmonized with their parents (4). Although the proportion of mothers choosing a date of birth in our sample was only over 10%, results of the regression model showed that this factor played a very important role in determining the cesarean section preference. Additionally, research showed that instrumental support was related to a preference for cesarean section. In the PICSS scale, instrumental support is defined as hand-on assistance with baby care or housework (43). Indeed, the health of pregnant women after cesarean delivery was significantly reduced compared to those who delivered vaginally, hence, it is difficult for them to guarantee the ability to conduct housework and childcare. A high level of instrumental support meant that it could increase the woman’s confidence in giving birth as well as preferring cesarean section in this delivery (43). Hence, it is difficult for them to guarantee the ability to conduct housework and childcare of pregnant women after cesarean delivery.

When evaluating preference for pain relief methods, study results showed that most women did not have any specific preference but mainly relied on physician’s advice. This finding is understandable when knowledge of pain relief methods is not common but requires careful consultation from health workers based on the condition and needs of the pregnant woman. Results of multivariate analysis showed that women who preferred cesarean section were more likely to choose pharmacological pain relief methods during childbirth compared to women who preferred vaginal delivery. This was appropriate when cesarean section is a complex process, and anesthesia measures should be used appropriately to minimize maternal pain and burden, as well as release the mothers’ anxiety related to negative birth experience (12, 44–46).

Our study also found that when evaluating mothers’ preference for pain relief approaches, around three-quarters of women indicated their need for non-pharmacological, and the most common method was support from partner/family members. This result was similar to several previous studies when indicating that 94% of women preferred this non-pharmacological pain relief approach (47), and support persons played the most important role in pain relief and significantly reduced labor time and analgesics’ need (48). This can be explained by the outstanding benefits of non-pharmacological pain relief methods compared to others as well as these benefits not only positively affected the mother during birth, but also offered them a positive experience with childbirth delivery and prepared them for new motherhood experience (14). In addition, childbirth delivery was a painful and lonely process, and having support from husbands or parents during labor helped pregnant women improve their self-efficacy and self-care ability to cope with labor pain (49). In Vietnam, the involvement of the support person in pain relief during childbirth has not been implemented widely due to concerns about hospital hygiene and infection. However, these barriers should be improved with holistic approaches, enabling pregnant women to be beneficial in minimizing their labor pain. We also found that the preference for the non-pharmacological method was driven by some factors such as the desire to have more children, finding health information from friends/relatives, and smartphone applications. In practice, smartphone applications are the common information sources for pregnant women to update current trends on pain relief methods during childbirth delivery. From that, they can consider different options and then choose preferable pain relief methods for themselves.

The findings of this study suggested several clinical and public health implications. First, pregnant women should be consulted on how to access and evaluate formal information in different sources, thereby increasing their knowledge about the benefits and risks of each delivery method, which assisted them in choosing the most appropriate method. Family members of pregnant women also need to be aware of these measures and avoid pressure in choosing the birth delivery method. Second, medical staff during routine antenatal care also need to offer reasonable advice and emphasize that the cesarean section is only suitable for medical reasons, as well as should not use the method to choose the time of giving birth according to pregnant women’s needs. Third, it is necessary to promulgate policies and regulations to limit cesarean sections for any reason other than medical reasons to reduce the rate of cesarean section. Fourth, obstetrics hospitals can consider developing and implementing a variety of non-pharmacological pain relief services during and after delivery. Acceptance and willingness to pay studies for these services should be performed in the future.

Our research had the strength of being conducted in two medical centers in different regions of Vietnam, which might increase the generalizability of our results. However, some limitations of the study should be noted. First, we used a convenient sampling method to recruit participants, leading to a decrease in the representativeness of our sample. Second, we used the cross-sectional study design for this study. This limited the ability to build causal relationships between preferences and related factors.

## 5 Conclusion

This study highlighted a high preference rate for cesarean section in urban pregnant women in Vietnam. Desire to have another baby, relative’s experience, preference for birth date, and instrumental social support were major drivers of the cesarean section preference. This preference was the important factor for the preference for pharmacological pain relief. Meanwhile, a high level of informational and emotional support was associated with non-pharmacological method preference. Holistic approaches from family, health facility, and policy should be performed to diminish the cesarean rate preference and promote the use of non-pharmacological pain relief methods during birth.

## Data availability statement

The original contributions presented in the study are included in the article/supplementary material, further inquiries can be directed to the corresponding author.

## Ethics statement

The studies involving humans were approved by The Institutional Review Board of the Hanoi Obstetrics and Gynecology Hospital approved the study protocol (Code: 07 QD/PS-TTĐT). Information about the objectives of the study, the participant's right to withdraw at any time, and contact information for members of the research group were completely provided to participants. Written informed consent was obtained from all study participants before recruitment and participation. All methods were carried out in accordance with relevant guidelines and regulations or the declaration of Helsinki. The studies were conducted in accordance with the local legislation and institutional requirements. The participants provided their written informed consent to participate in this study.

## Author contributions

TN: Conceptualization, Data curation, Formal analysis, Software, Supervision, Writing – original draft, Writing – review & editing. LN: Conceptualization, Formal analysis, Software, Writing – original draft, Writing – review & editing. HN: Data curation, Methodology, Supervision, Writing – original draft, Writing – review & editing. VD: Data curation, Investigation, Software, Writing – original draft, Writing – review & editing. TV: Conceptualization, Formal analysis, Methodology, Writing – original draft, Writing – review & editing. CL: Formal analysis, Methodology, Writing – review & editing. MZ: Methodology, Writing – original draft, Writing – review & editing. RH: Conceptualization, Methodology, Supervision, Writing – review & editing. CH: Conceptualization, Methodology, Writing – review & editing.
